# Free Wall Myocardial Rupture and Ventricular Septal Defect in an Ambulatory Patient

**DOI:** 10.7759/cureus.8470

**Published:** 2020-06-06

**Authors:** Waqas Memon, Muhammed Aamir, Areeka Memon, Maha Mikhail

**Affiliations:** 1 Internal Medicine/Nephrology, Virginia Commonwealth University, Richmond, USA; 2 Internal Medicine, Carle Foundation Hospital, Urbana, USA; 3 Osteopathic Medicine, Edward Via College of Osteopathic Medicine, Blacksburg, USA; 4 Cardiology, Carle Foundation Hospital, Urbana, USA

**Keywords:** myocardial rupture, cardiology, critical care, rare presentation, myocardial infarction

## Abstract

Myocardial rupture is a rare complication of acute myocardial infarction (MI), usually presenting with chest pain. The most common site of rupture is the anterior wall. Myocardial rupture presents similar to cardiac tamponade, most frequently as cardiogenic shock. Many clinical conditions, however, present similarly. The differential diagnosis should include myocardial rupture if clinical suspicion is high. This report describes a 77-year-old man with a medical history putting him at significant risk for coronary artery disease status, including a coronary artery bypass graft, chronic kidney disease stage 3, and hyperlipidemia. He presented at the ED for worsening shortness of breath and feeling unwell. Transthoracic echocardiography revealed an anterior, anterolateral akinesis, a ventricular septal defect, and free wall rupture. Myocardial rupture is an acute cardiac emergency; a high level of clinical suspicion may help in detecting this rare manifestation of acute MI.

## Introduction

Myocardial rupture is a rare and serious complication of acute myocardial infarction (MI), usually presenting as an acute cardiac emergency. The most common symptom on presentation is chest pain, and the most common site of rupture is the anterior wall [1-2]. The overall incidence of free wall rupture is declining, from >4% in 1977-1982 to <2% in 2001-2006, a decrease attributed to the advances in reperfusion therapies and better clinical awareness of blood pressure goals [3].

## Case presentation

A 77-year-old man with a past medical history putting him at significant risk for coronary artery disease, including a coronary artery bypass graft, chronic kidney disease stage 3, and hyperlipidemia presented at the ED for worsening shortness of breath and feeling unwell. An initial examination showed that the patient was hypotensive and tachycardic, with 98% oxygen saturation on room air. He was positive for diffuse crackles over the right and left hemithorax without any focal neurological findings. Cardiac examination showed that he was positive for jugular vein distention and trace lower extremity edema (Table 1).

**Table 1:** Laboratory tests. BNP, brain natriuretic peptide

Laboratory test	Actual value	Normal value
Lactic acid	7.28 mg/dL	0.4-2.0 mg/dL
Troponin	0.88 ng/mL	<0.4 ng/dL
BNP	>35000 pg/mL	<100 pg/mL
Procalcitonin	0.14 ng/dL	<0.10 ng/dL

Because of high clinical suspicion of impending shock, the patient was started on broad-spectrum antibiotics and norepinephrine for vasopressor support. He became hypoxic and required bilevel positive airway pressure and vasopressor support. Transthoracic echocardiography revealed anterior, anterolateral akinesis, a ventricular septal defect, and free wall rupture, findings confirmed by both echo/contrast echo and transesophageal echocardiography (TEE) showing contrast in the pericardial sac (Videos 1-2).

**Video 1:** Transthoracic echocardiogram depicting severely reduced LV systolic function with regional wall motion abnormalities. The anterior wall was severely hypokinetic, and the anterolateral wall was akinetic with scarring and wall thinning. A contained LV free wall rupture at the mid-cavity level was noted. Additionally, a large ventricular septal defect was seen in the mid and apical segment of the myocardium. The right ventricle was dilated and severely hypokinetic. Biatrial enlargement was present. LV, left ventricular

**Video 2:** Transthoracic echocardiogram at the mid esophageal level showing the short axis of the LV with confirmation of free wall rupture of the anterolateral wall of the LV at the mid cavity with ventricular septal defect. LV, left ventricle

The patient was switched to dobutamine for inotropic support, and emergency cardiovascular surgery was considered. The patient continued to do poorly, requiring increased vasopressor and ventilator support. After consultation with his family, the patient was made comfortable and died.

## Discussion

Myocardial rupture presents similar to cardiac tamponade, most frequently as cardiogenic shock resulting in sudden cardiac death [2-4]. Without proper imaging and/or an autopsy, death in these patients can be attributed to fatal arrhythmia, heart block, or massive pulmonary embolism [5]. Determining proper treatment is difficult because many clinical conditions share similar presentations. Thus, the differential diagnosis of these patients should include myocardial rupture if clinical suspicion is high.

Myocardial rupture can be diagnosed by standard TEE, although contrast-enhanced echocardiography can also be helpful. Cardiac MRI can also help confirm an impending myocardial rupture. Management depends on early recognition of myocardial rupture, with immediate medical therapy including the administration of intravenous fluids, inotropes agents, and vasopressors to maintain patient hemodynamics and prevent or reduce shock [6]. Cardiothoracic surgery is the definitive treatment [7-9].

## Conclusions

Early diagnosis of myocardial rupture early is extremely important in preventing catastrophic outcomes. Myocardial rupture should be included in the differential diagnosis of patients presenting with acute MI. Early recognition, stabilization, and emergency surgical intervention can improve patient survival.
